# Research Progress on the Structure and Function of G3BP

**DOI:** 10.3389/fimmu.2021.718548

**Published:** 2021-08-30

**Authors:** Weifang Kang, Yue Wang, Wenping Yang, Jing Zhang, Haixue Zheng, Dan Li

**Affiliations:** State Key Laboratory of Veterinary Etiological Biology and OIE/National Foot and Mouth Disease Reference Laboratory, Lanzhou Veterinary Research Institute, Chinese Academy of Agricultural Sciences, Lanzhou, China

**Keywords:** G3BP, stress granules, virus proliferation, translation regulation, cancer

## Abstract

Ras-GTPase-activating protein (SH3 domain)-binding protein (G3BP) is an RNA binding protein. G3BP is a key component of stress granules (SGs) and can interact with many host proteins to regulate the expression of SGs. As an antiviral factor, G3BP can interact with viral proteins to regulate the assembly of SGs and thus exert antiviral effects. However, many viruses can also use G3BP as a proximal factor and recruit translation initiation factors to promote viral proliferation. G3BP regulates mRNA translation and attenuation to regulate gene expression; therefore, it is closely related to diseases, such as cancer, embryonic death, arteriosclerosis, and neurodevelopmental disorders. This review discusses the important discoveries and developments related G3BP in the biological field over the past 20 years, which includes the formation of SGs, interaction with viruses, stability of RNA, and disease progression.

## Introduction

G3BP binds to the SH3 domain of the Ras-GTPase activating protein (GAP) in serum-stimulated cells, as first reported by Paker in 1996 (1). The homologue of G3BP in Drosophila is known as Rasputin, encoded by the Rin gene (2). G3BP-like proteins have also been identified in plants such as Arabidopsis thaliana (3). In fission yeast Nxt3 is a G3BP homologue (4). However, Annibaldi et al. have suggested that there is no interaction between G3BP1 and Ras-GAP, and the role of G3BP1 in the Ras signaling pathway needs to be further explored (5). The G3BP family also exhibits important interactions with other signaling pathways. This review discusses the effects of these interactions in the context of disease. The G3BP family consists of three homologous proteins, namely, G3BP1, G3BP2a and G3BP2b. G3BP1 and G3BP2 are encoded by different genes on human chromosomes 5 and 4 and mouse chromosomes 11 and 5, respectively. G3BP2b is a spliced ​​isoform of G3BP2a that lacks 33 amino acids in its central region (6). G3BP, a site-specific ribonucleic acid endonuclease, is expressed in all normal cells. G3BP1 is highly expressed in the lungs and kidneys, while G3BP2 is highly expressed in the small intestine and brain (3, 6). G3BP may be an important downstream effector of the Ras signaling pathway and may affect the RNA metabolism in a Ras-GAP-dependent manner. G3BP is closely related to the formation and activity of stress granules (SGs). G3BP1 can be used as an antiviral factor to promote the retinoic acid-inducible gene I (RIG-I) to recognize RNA viruses (7, 8). It can also enhance the binding of cyclic GMP-AMP synthase (cGAS) and DNA by promoting the formation of a large cGAS complex (9). Some viruses have evolved different mechanisms of action by manipulating G3BP to facilitate their replication and evade the cellular immunity of the host. G3BP is involved in a variety of disease processes, including cancer invasion and metastasis, and virus survival in the host. Currently, there is a lot of evidences indicating that targeting G3BP is a potential therapeutic strategy for cancer. This review discusses the role of G3BP in regulating the composition of SGs, interacting with viruses, regulating the stability of RNA, and various disease processes.

## Structure of G3BP

p120-RasGAP is a 120-kDa cytosolic protein and has an amino-terminal region containing an Src Homology 3 (SH3) domain flanked by two SH2 domains (10). The C-terminus contains a GAP domain that catalyzes the activation of Ras by hydrolyzing GTP-bound active Ras to the inactive GDP-bound form of Ras. The p120-RasGAP SH3 domain is essential for downstream signaling of Ras (11). p120-RasGAP protein may bind to G3BP protein (GAP SH3-binding protein) through its SH3 structural domain (1).

**Figure 1:**
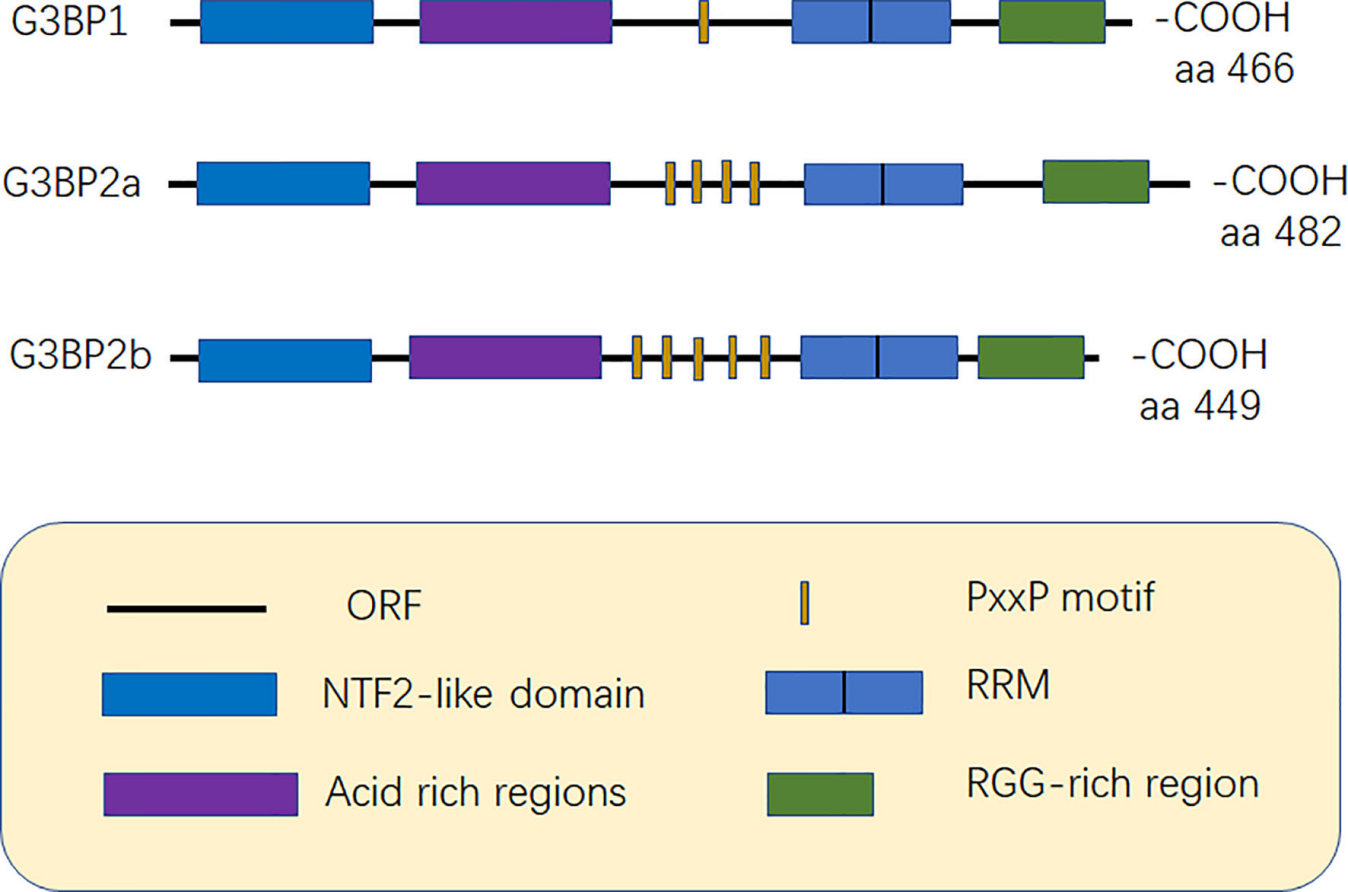
Structure of G3BP. G3BP1 contains five distinct motifs, nuclear transport factor 2 (NTF2) domain, Acidic-rich region, proline-rich (PxxP) motif, RNA recognition motif (RRM), and RGG domain (arginine -glycine-rich boxes), at the C-terminal. G3BP1 and G3BP2 can be distinguished according to the number of PxxP motifs as G3BP1 has one PxxP motif, while G3BP2a and G3BP2b have four and five PxxP motifs, respectively (6, 12).

G3BP contains five distinct motifs, nuclear transport factor 2 (NTF2 domain), acidic rich region, proline-rich (PxxP) motif, RNA recognition motif (RRM), and RGG domain (arginine-glycine-rich boxes), at the C-terminal (12) (**Figure 1**).

The NTF2 structural domain forms a homodimer with each chain consisting of three alpha helices and one beta-sheet (13). The crystal structure of the NTF2 domain of Rasputin, which shares 54.2% and 55.6% sequence identity with the NTF2 domain of human G3BP1 and G3BP2a, respectively (14). The peptide containing the FGDF motif binds to a hydrophobic groove on the surface of the NTF2 domain of G3BP, which inhibits the formation of SGs (15). The NTF2 domain is associated with nuclear transport and cellular localization and can also be used as a carrier to mediate its oligomerization as well as that of other chaperone proteins (13, 16). The phosphorylation of G3BP at serine (Ser-149) also inhibits its oligomerization, thus limiting the assembly of SGs (16); however, a recent study by Panas et al. contradict this report as the authors believe that phosphorylation at the Ser-149 site has no effect on the formation of SGs (17). Phosphorylation-mediated modification of Ser-149 affects the role of G3BP in mediating-mRNA decay (18). The presence of NTF2 domain also plays an important role in viral replication because it can bind to the viral motifs and be recruited by the viral replication complex (19).

Acidic rich region can negatively regulate phase separation (20). The PxxP domain of G3BP is required for the activation of protein kinase (PKR), antiviral activity of G3BP1, and nucleation of SGs (8, 21). G3BP1 and G3BP2 can be mainly distinguished based on the number of PxxP motifs they possess. G3BP1 has only one PxxP motif, which may limit its ability to interact with proteins, while G3BP2a and G3BP2b have four and five PxxP motifs, respectively (3, 6) (**Figure 1**).

The RRM domain includes two short sequences, RNP1 and RNP2, which consist of conserved hydrophobic amino acids distributed throughout the motif that are essential for RNA binding (12). The most significant difference between the G3BP1 and G3BP2 RRM is the substitution of Ile for Val in the RNP-2 common sequence (6). RGG is composed of arginine-glycine-glycine clusters and is hence named RGG. The RGG domain is also easily methylated because it contains a large amount of arginine. The methylation of G3BP1 inhibits SGs formation (22). The RG-rich domain of G3BP1 increases its binding affinity to RNA and mediates protein-protein interactions, thereby stabilizing the G3BP1-RNA complex (20). The RG-rich domain is also associated with nucleocytoplasmic shuttling (23). The RGG domain of G3BP is essential for the recruitment of the host translation machinery. For example, replication of the Semliki Forest virus (SFV) is reduced in the cells of the G3BP mutants that lack the RGG domain (24).

## G3BP and SGs

### Formation of SGs

**Figure 2:**
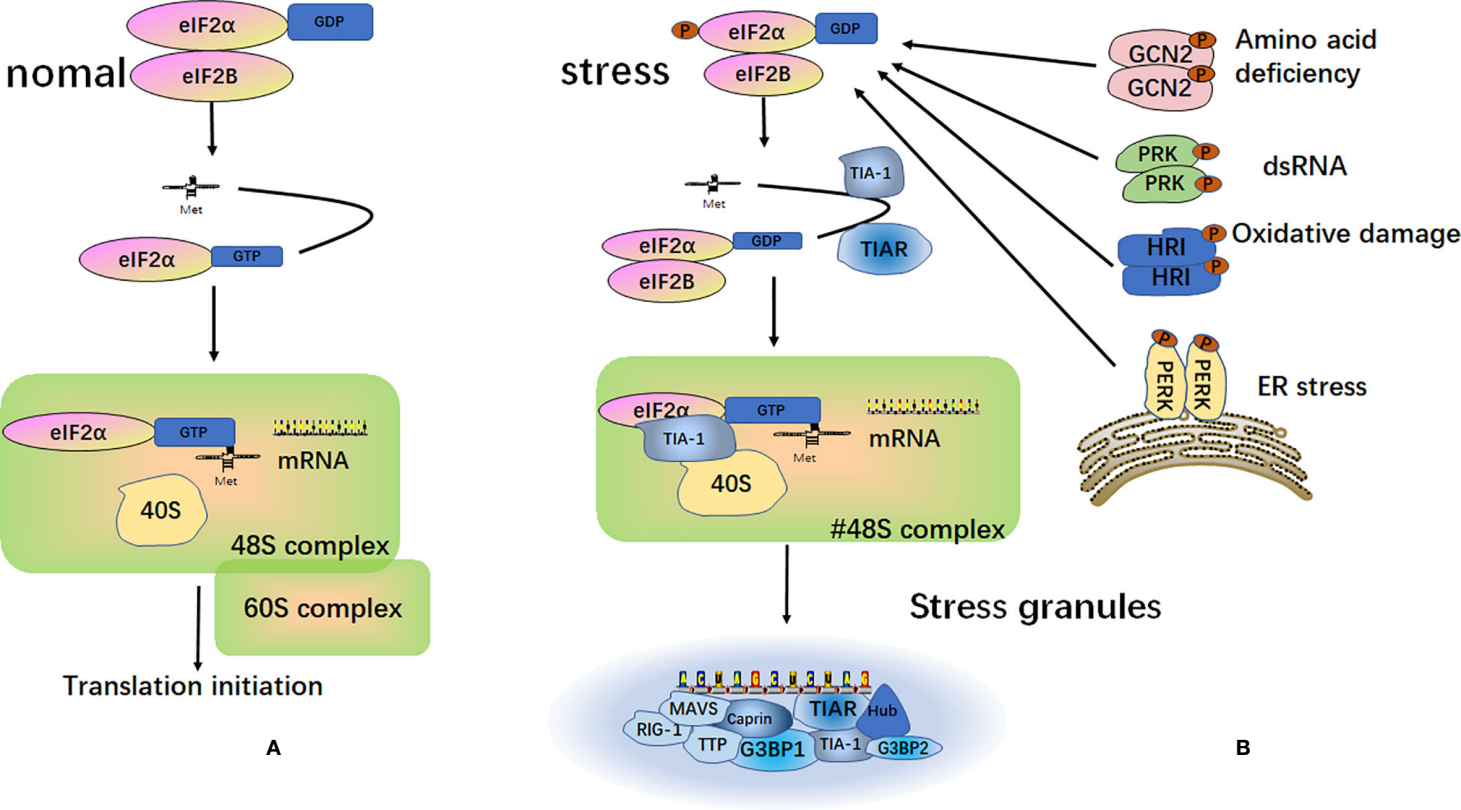
Formation of stress granules. **(A)** Under normal circumstances, after the separation of the eukaryotic translation initiation factor 2 subunit alpha (eIF2α) and eukaryotic translation initiation factor 2B (eIF2B), eIF2α and tRNA^Met^ form a ternary complex called eIF2-GTP-tRNA^Met^. This complex binds to mRNA and the 40S and 60S ribosomal subunits to participate in the process of translation; **(B)** Under different external stimulus conditions, four kinases can be activated to induce phosphorylation of the α subunit of eIF2. The phosphorylated state of eIF2 is stabilized by binding to eIF2B. This binding prevents eIF2B from catalyzing GDP into GTP. This reduces the level of eIF2-GTP-tRNA^Met^ ternary complex and does not allow the formation of a functional 48S initiation complex. Instead, a non-classical 48S initiation complex is produced under the action of TIA-1 and TIAR. This complex cannot recruit the 60S ribosomal subunit to participate in translation, but it is recruited into the stress granules.

SGs are particles with a high phase density in the cytoplasm of eukaryotic cells. SGs are aggregates of translationally stalled membrane-less messenger ribonucleoprotein complexes (mRNPs). When eukaryotic cells are exposed to environmental stresses (e.g., heat, oxidative conditions, viral infections, hyperosmolarity, and UV irradiation), RNA binding proteins (RBP) mediate their condensation to form SGs by recruiting the mRNPs (25–27). The classical assembly pathway of SGs is mainly mediated by the phosphorylation of eukaryotic translation initiation factor 2 subunit alpha (eIF2α). The elF2α kinase family includes PKR endoplasmic reticulum kinase (PERK/PEK) (28), protein kinase R (PKR) (29), general control nonderepressible 2 (GCN2) (30), and heme-regulated inhibitor (HRI) (31, 32). Furthermore, eIF2α is phosphorylated by one or more of these kinases, thereby reducing the concentration of the elF2-GTP-tRNA^Met^ ternary complex. When the level of the ternary complex is reduced, the related RNA binding proteins, T-cell intracellular antigen-1 (TIA-1) and T‐cell restricted intracellular antigen‐related protein (TIAR), promote the assembly of the atypical initial 48S complex. This complex cannot recruit the 60S ribosomal subunit to participate in translation but can be recruited into SGs to participate in its assembly (25, 33, 34) (**Figure 2**).

The components of SGs mainly include translation initiation factors eukaryotic translation initiation factor 4G (eIF4G), eukaryotic translation initiation factor 3 (eIF3), and eukaryotic translation initiation factor 5A (eIF5A) (35), small ribosomal subunits, and various aggregates of mRNA-binding proteins, such as the cytoplasmic activation/proliferation-associated protein-1 (Caprin1), tristetraprolin (TTP) (36–38), TIA-1, TIAR (25, 39) and G3BP (40). TIA-1 is an RNA-binding protein consisting of three RRM and a prion-related domain. It binds to the eIF2α-deficient preinitiation complex and induces the aggregation of mRNPs to form the SGs (41, 42). The Roquin family proteins can trigger the accumulation of mRNA and allow it to enter the SGs to limit the use of energy and nutrients (43). Formation of SGs regulates stress response, viral infection and signaling pathways, which are closely related to cell apoptosis and nuclear function (44). SGs can also act as an oxidative regulator in cells under stressful conditions (45). When host cells are infected by viruses, SGs can resist viral infection by isolating the invader and limiting the translation of viral mRNAs. However, many viruses have also evolved mechanisms to inhibit the formation of SGs and evade the host antiviral response. Some viruses also target SGs to make favorable choices. SGs are the downstream participants in the cellular stress response. Their components, G3BP1 and G3BP2, may play important roles in viral replication and may also promote the innate immune response after viral infection of the host cell.

### G3BP Is a Component of SGs

Both G3BP1 and G3BP2 are recruited to SGs in various human cells (46). G3BP1 is distributed in the cytoplasm and forms SGs under stressful conditions (16, 20). G3BP2 and G3BP1 can also form homo- or heterodimers to induce the formation of SGs (46). P21^ras^ affects the recruitment of G3BP to SGs in a time-dependent manner (16). G3BP is expected to be disordered to a large extent, which is a common feature of SGs. There are three different intrinsically disordered regions (IDRs) in G3BP1 and their interactions regulate the liquid-liquid phase separation of SGs (47). The overall number of early SGs is related to the expression of G3BP1, and overexpression of G3BP increases the formation of SGs (16, 48, 49). SGs assembly can also be mediated by the heterogeneous phase separation of G3BP1 and RNA (20). The carboxyl (C)-terminus region of G3BP can induce the phosphorylation of eIF2α, which can further induce the assembly of SGs (50). Methylation, phosphorylation and acetylation of G3BP influence the formation of SGs. It has also been indicated that large G3BP proteins induce SGs formation independent of the phosphorylation of eIF2α (49).

### G3BP Interacts With Host Proteins to Promote the Formation of SGs

G3BP regulates SGs formation by binding to different proteins. The pseudophosphatase MK-STYX (mitogen-activated protein kinase phosphoserine/threonine/tyrosine-binding protein) interacts with G3BP1 to dephosphorylate it at Ser-149 and induce the formation of SGs (51). Poly(ADP-ribose) (pADPr) interacts with G3BP to regulate nuclear translocation and promote the assembly of cytoplasmic SGs (23). Ubiquitin associated protein 2-like (UBAP2L) is necessary for the nucleation of SGs. It acts upstream of G3BP and promotes the formation of G3BP1 core and the assembly and growth of SGs (52). Jumonji C domain-containing protein 6 (JMJD6) is a novel component of SGs that interacts with the G3BP1 complex and its expression reduces G3BP1 methylation and promotes SGs formation (22). Transactive response DNA-binding protein-43 (TDP-43) is a multifunctional protein that regulates the transcription of G3BP, aggregation of TIA-1, and assembly of SGs (53). Tudor-staphylococcal nuclease (Tudor-SN), also known as p100 or staphylococcal nuclease (SN) domain-containing protein 1, is a novel SGs component. The SN domain of Tudor-SN can interact with G3BP, which is then recruited into the SGs (54). The acetylation of G3BP1 is regulated by histone deacetylase 6 (HDAC6) and CBP/p300, and it promotes the breakdown of SGs (55). Cytoplasmic phosphoprotein (Caprin) also binds to G3BP to promote SGs formation (36). K08F4.2, which is the C. elegans orthologue of the mammalian G3BP (79% amino acid homology), elegans SIR-2.4 interacts with K08F4.2/G3BP and localizes to SGs (56).

### G3BP Interacts With Host Proteins to Inhibit the Formation of SGs

The testis-specific protein (melanoma-associated antigen gene B2) and DEAD-box decarboxylase 5 (DDX5) can inhibit SGs formation by inhibiting the translation of G3BP, which increases the tolerance of cells to stress (48). G3BP1 is methylated by protein arginine methyltransferase 1 (PRMT1), PRMT5, and low-density lipoprotein receptor-related protein 6 (LRP6), which inhibits the formation of SGs (22, 57). The FGDF motif of the mammalian ubiquitin-specific protease (USP10) binds to G3BP, thereby affecting the interaction of G3BP1/2 and Caprin1 to inhibit the formation of SGs (36). Casein kinase 2 (CK2) phosphorylates the Ser-149 site of G3BP1 *in vitro* and *in vivo*, inhibits the formation of SGs, and promotes protein synthesis (58).

## G3BP Inhibits Viral Replication

### G3BP Inhibits the Replication of DNA Virus

G3BP1 enhances the binding capacity of cGAS and DNA by promoting the formation of a large cGAS complex, which then plays an important positive regulatory role in the DNA-induced type I interferon (IFN) pathway. Therefore, G3BP1 participates in the cGAS-mediated antiviral response and plays an important role in resisting DNA virus infection (9). Many DNA viruses use various mechanisms to evade host defenses. For example, The Cap protein of PCV3 inhibits the interaction between cGAS and G3BP1 and affects the production of IFN-β. Overexpression of G3BP1 reduces the inhibitory effect of Cap on the cGAS-STING signaling pathway (59) (**Table 1**).

**Table 1:** G3BP inhibits viral replication.

Genome	Virus	Mechanism of Action	References
ssDNA	PCV3	The capsid protein of PCV3 interacts with G3BP1 to prevent DNA recognition by cGAS and inhibit the production of interferon	(59)
dsRNA	MRV	μNS interacts with G3BP1 and interferes with the formation of SGs	(60, 61)
(+) ssRNA	PEDV	G3BP1 exerts antiviral effects, the over expression of G3BP1 reduces the replication of PEDV	(62)
	FMDV SVV EV71 EMC	3A interacts with G3BP1, upregulates LRRC25 and inhibits RLH signaling	(63)
	FMDV	G3BP1 interacts with FMDV IRES and negatively regulates translation	(64)
		3C^pro^ and L^pro^ cleaves G3BP	(64, 65)
	PRRSV	N protein interacts with G3BP1 and induces G3BP1 phosphorylation which loses the antiviral effect of G3BP1	(66)
	ERAV	L^pro^ cleaves G3BP1	(67)
	FCV	NS6^pro^ cleaves G3BP1	(68)
	CVB3 PV	3C^pro^ cleaves G3BP1	(69, 70)
	EMCV	G3BP1 was cleaved	(71)
	SVV	G3BP1 enhances the virus-induced NF-κB signaling pathway	(72)
		3C^pro^ disrupts the interaction of eIF4GI-G3BP1	(72)
	EV71	2A protein, 2A^pro^ and L protein disrupt the interactions of eIF4GI-G3BP1	(73, 74)
(-)ssRNA	SeV VSV	G3BP1 forms a complex with RNF125 and RIG-I to promote the expression of RIG-I	(75)
ssRNA-RT	HIV-1	Gag protein, eEF2 interact with G3BP1 to inhibit SGs	(76)
		Viral RNA interacts with G3BP1 and restricts viral translation	(77)

### G3BP Inhibits RNA Virus Replication

G3BP antagonize viral growth. Therefore, the viral proteins will target G3BP for cleavage, inhibiting the formation of SGs and evading natural immunity. In some cases, G3BP also increased cytokine production during viral infection, thereby modulating the immune response.

#### G3BP Interacts With Viral Proteins

G3BP1 exhibits antiviral effects and inhibits the replication of porcine epidemic diarrhea virus (PEDV) (62). G3BP1 interacts directly with three independent sequences of the foot-and-mouth disease virus (FMDV) internal ribosome entry site (IRES), which changes the structure of the IRES. It also interacts with the polypyrimidine tract-binding (PTB) and eukaryotic translation initiation factor 4B (eIF4B), through its C-terminal domain, thus to negatively regulate translation, and affect IRES-dependent and cap-dependent translation (64). The non-structural proteins σNS and μNS of mammalian reovirus (MRV) interact with G3BP1 and interfere with SGs formation. MRV replication is enhanced in cells with G3BP1 knockdown (61). It has also been reported that MRV can induce the formation of SGs in the early stage of infection that is dependent on the phosphorylation of eIF2α, which promotes virus replication (60). Although the phosphorylation level of elF2α is still high in the late stage of MRV infection, the level of SGs formation is reduced (78). Infection of cells with porcine reproductive and respiratory syndrome virus (PRRSV) induces the formation of SGs, which are associated with the viral replication complex (VRC); however, G3BP1 does not play a role in PRRSV replication. The PRRSV N protein phosphorylates G3BP1 and abolishes its antiviral effect. Moreover, the knockout of G3BP1, G3BP2, and USP10 does not affect viral replication (66).

#### G3BP Is Cleaved by the RNA Viral Protein

G3BP1 is an antiviral protein (8). G3BP is cleaved by viral proteins, which destroys the assembly of cytoplasmic SGs and manipulates the stress response pathway and innate antiviral response. The 3C protease (3C^pro^) (64) and lead protease (L^pro^) (65)of FMDV cleave G3BP1 at glutamate-284 (E284). The L^pro^ of equine rhinitis A virus (ERAV) and NS6^pro^ of feline calicivirus (FCV) (68) promote the cleavage of G3BP at E405/V406 sites, thereby inhibiting the formation of SGs and facilitating the evasion of the host natural immunity (67). The 3C^pro^ of coxsackie virus type B3 (CVB3) (69), human enterovirus D68 (EV-D68) (79) and poliovirus (PV) can cleave G3BP1 at Q325, leading to disassemble of the formation of SGs, which contributes to viral translation. However, the Q326E mutation in G3BP1 can resist cleavage by 3C^pro^ (70).

#### G3BP Promotes RIG-I to Recognize RNA Viruses

When infected by RNA viruses, cells produce antiviral stress granule (avSGs) in a PKR-dependent manner. These avSGs contain RIG-I and G3BP and recognize non-self RNA, which is essential for triggering antiviral response. RIG-I binds to the mitochondrial membrane protein IFN promoter stimulator-1 (IPS-1), promotes RIG-I to recognize RNA viruses, and promotes the activation of type I IFN responses. Knockout of G3BP impairs avSGs formation and IFN-α gene activation (7). Small ribonucleic acid viruses, such as the Seneca valley virus (SVV), enterovirus 71 (EV71), encephalomyocarditis virus (EMCV), and FMDV have 3A proteins that degrade G3BP1 by upregulating the expression of the autophagy-associated protein leucine-rich repeat-containing protein 25 (LRRC25), inhibiting the expression of RIG-1 and MDA5, evading innate immunity, and increasing viral replication (63). G3BP1 inhibits the replication of Sendai virus (SeV) and vesicular stomatitis virus (VSV). G3BP1 forms a complex with Really Interesting New Gene finger protein 125 (RNF125) and RIG-I, thereby decreasing RNF125 level through auto-ubiquitination. This promotes the expression of RIG-I and positively regulates antiviral innate immunity (75).

#### G3BP1 Promotes the Production of Cytokines

G3BP1 promotes activation of innate immune transcriptional responses *via* the nuclear factor-κB (NF-κB) and c‐Jun N‐terminal kinase (JNK) pathways (8). G3BP1 is essential for activating the NF-κB signaling pathway induced by SVV. G3BP1 Knockdown reduces the mRNA levels of interleukin-6 (IL-6) and tumor necrosis factor-alpha (TNF-α). G3BP1 interacts with PKR in SGs, and they regulate each other’s activities (8). SVV induces the formation of transient SGs (tSGs) through eIF2α phosphorylation in a PKR-dependent manner. Although the formation of tSGs has no effect on the replication of SVV, G3BP1, as a key factor of SGs, plays a critical role in the activation of the NF-κB signaling pathway induced by SVV, which activates the antiviral response of the body (72). ORF120, an early-late Orf virus (ORFV) encoded protein, is capable of positively regulating NF-κB signaling by interacting with G3BP1 (80). When infected by EMCV, G3BP1 gets cleaved and dephosphorylates PKR. This leads to impaired formation of SGs induced by EMCV and a decrease in IFN-β gene activation, thereby weakening the activity of antiviral cytokines (71). EV71 also relies on PKR to recognize the viral dsRNA, which activates the PKR-eIF2α signaling cascade and leads to the formation of tSGs. Unlike SVV, cells and viral mRNAs are isolated in tSGs after EV71 infection, which leads to the overall shutdown of translation to inhibit viral translation. However, SVV 3C^pro^ (72) and Enterovirus 71 (EV71) 2A protein, 2A^pro^ and L protein can block the formation of tSGs by disrupting the eIF4GI-G3BP1 interaction and inducing the formation of aSGs. The formation of aSGs is beneficial for virus translation (73, 74).

#### G3BP Inhibits the Replication of Retroviruses

The role of G3BP1 in human immunodeficiency virus (HIV) infection is complex. HIV type 1 (HIV-1) RNA specifically interacts with G3BP1 in the cytoplasm, which isolates viral transcripts, thereby preventing the translation or packaging of viral proteins. This restricts mRNA translation, viral protein production, and viral particle formation (77). However, the capsid domain of the HIV-1 Gag protein can also interact with G3BP1, replacing the eukaryotic elongation factor 2 (eEF2) and disassembling the preformed SGs. This response may be beneficial for virus replication (76). HCV-1 nucleocapsid protein (NC) induces the production of SGs containing TIAR1, G3BP1, eIF3, and poly (A)-binding protein. These SGs are not depolymerized by the previously mentioned Gag protein. The host factor Staufen1 inhibits the NC-induced production of SGs (81).

#### Viruses Inhibit the Formation of SGs

To escape the resistance of host cells, most viruses have evolved various strategies to inhibit the formation of SGs and promote viral replication (**Table 2**).

**Table 2:** Virus Inhibits the formation of SGs.

Genome	Virus	Mechanism of action	References
dsDNA	PNYDV ABMV	AtG3BP binds to nuclear shuttle protein and inhibits the formation of SGs	(3)
	HSV-1	ICP8 protein binds G3BP and blocks the assembly of SGs	(82)
(+) RNA	SARS-CoV-2	N protein interacts with G3BP, decomposition SGs	(83)
	JEV	G3BP and USP10 are isolated and inhibit the formation of SGs	(84)

In plants, *Arabidopsis* processes a G3BP-like protein (AtG3BP) containing the NTF2-RRM domain, which binds to the FVSF motif of the nuclear shuttle protein of abutilon mosaic virus (ABMV) and the FNGSF motif of the nuclear shuttle protein of pea necrotic yellow dwarf virus (PNYDV), thereby inhibiting the function of SGs (3). The FGDF motif is found at the C-terminus of the herpes simplex virus (HSV-1) ICP8 protein, which binds to G3BP1/2 and blocks SGs assembly. This FGDF motif is also found in SFV and USP10, which can also bind to G3BP1/2 to block the assembly of SGs (82). IDR1 in the N protein of the severe acute respiratory syndrome coronavirus 2 (SARS-CoV-2) interacts with G3BP1/2 to decompose SGs and promote the assembly and production of the virus (83). Katoh et al. reported that the core protein of Japanese encephalitis virus (JEV) interacts with Caprin-1 to recruit G3BP and USP10 to the perinuclear region. This inhibits the production of SGs which promoting the reproduction of the virus (84). However, so far, there is no scientific evidence that G3BP exhibits antiviral effect in these viruses or that it is the only scaffold protein of SGs, which would suggest that the virus exerts antiviral effects by targeting G3BP to inhibit the production of SGs.

## G3BP Promotes Virus Replication

### G3BP Promotes the Replication of DNA Virus

G3BP1 promotes virus proliferation (**Table 3**). Hence, it can be used as a proviral factor that plays important role in different replication cycles and structures of the virus (96). Vaccinia virus (VV) is different from other DNA viruses because it only replicates in the cytoplasm. The replication and assembly of the viral genome occurs in the cytoplasmic region of the DNA virus factory (97). VV can use G3BP to act at different stages of replication. In the early stage of infection, the virus factory is surrounded by the rough endoplasmic reticulum, which recruits key translation initiating factors, such as G3BP1 and Caprin-1. These molecules increase the activities of viral RNA polymerase and transcription factors, participate in the transcription of intermediate genes, and regulate the transition from early to late replication (85). However, when host cells are infected with VV lacking the E3L mutant protein, avSGs surround the VV replication factory and gather around the infected cell. The avSGs include SGs-related proteins, such as G3BP1/2, TIA-1 and USP10, which have the ability to resist viral replication (98).

**Table 3:** G3BP promotes viral replication.

Genome	Virus	Mechanism of Action	References
dsDNA	VV	G3BP1,Caprin-1 is recruited to the viral plant and enhances the transcription of VV	(85)
dsRNA	IBDV	The overexpression of G3BP1 enhances SGs formation and promotes viral replication	(86)
(+)ssRNA	CHIKV	G3BP1/2 regulates the conversion of viral genome to negative strand synthesis	(87)
	SFV	G3BP1 binds to nsP3 protein and 40S ribosomal subunit to promote efficient translation of viral mRNA	(24)
	EEEVVEEV	FXR interacts with G3BP1/2 and nsP3 proteins to assemble the viral replication complex (vRC)	(88)
	HCV	G3BP1 acts as a component of the replication complex to control the replication of HCV	(89)
	DENV2	SfRNA interacts with G3BP1, G3BP2, Caprin1 and regulates the translation of ISGs mRNA	(90, 91)
	RUBV	G3BP co-localizes with viral ssRNA and participates in the role of viral replication	(92)
	NDV	G3BP1 regulates NDV replication through controlling the translation	(93)
	ZIKV	Virus interacts with G3BP1 to impair the assembly of SGs	(94)
(-) ssRNA	RSV	SG is produced during RSV infection to enhance replication	(95)

### G3BP Promotes the Replication of RNA Virus

G3BP plays the role of proviral factor in alphaviruses. It interacts with the viral non-structural protein 3 (nsP3) to promote the effective translation of viral mRNA. Chikungunya virus (CHIKV) also relies on G3BP1/2 for efficient replication, and knockdown of G3BP1/2 inhibits its replication. In the early stage of infection, the FDGF motif of nsP3 forms aggregates with G3BP1/2, which can regulate the conversion of the viral genome to synthesize the negative strand. G3BP1/2 may also be involved in stabilization of naked viral RNA. Later in the replication cycle of CHIKV, the virus induces the formation of SGs, which are different from true SGs because they lack SGs markers, such as TIA-1, TIAR, eIF3 (87). After G3BP is combined with CHIKV HVD, its HVD motif is divided into upstream and downstream. Then the nucleosome assembly protein 1 (NAP1) family members interact with the two motifs located upstream and downstream, and play a stimulating function in virus replication (99). The NTF2 and RGG domains of G3BP1 bind to the nsP3 and 40S ribosomal subunit of SFV, respectively, to aggregate the viral replication complex and recruit translation initiation factors, thereby promoting the efficient translation of viral mRNA (19, 24, 100). The hypervariable domain (HVD) of the eastern equine encephalitis virus (EEEV) and Venezuelan equine encephalitis virus (VEEV) nsP3 protein interacts with RNA-binding protein farnesoid X receptor (FXR) and G3BP1/2 to assemble the VRC, which effectively increases their replication efficiency (88, 101). G3BP1 and G3BP2 are essential for regulating the replication of Sindbis virus (SINV) *in vivo*. There are potential interaction between nsp2, nsp3, nsp4 and G3BP1/2. G3BP1/2 interacts with nsP3 and nsP4 to limit the expression of SINV polyprotein, which may be beneficial for virus replication, or on behalf of the host cell to restrict virus replication (102).

G3BP1 may be a component of the hepatitis C virus (HCV) replication complex. G3BP1 may directly bind to the non-structural protein 5B (NS5B) or RNA of HCV to control its replication by regulating the components of the HCV replication complex (89). HCV triggers the appearance of SGs in a PKR-and IFN-dependent manner. G3BP1 and TIA-1 are essential components for the efficient assembly of infectious virus particles, and TIAR is necessary for the release of infectious virus particles. They regulate HCV infection by affecting the early and late stages of the HCV life cycle (103). G3BP1 controls the translation of interferon-stimulated genes (ISGs) (104). When infected with dengue virus (DENV) types 2 and 3, human lung pneumocyte cells (A549) cells recruit G3BP1. The DEMV2 non-coding subgenomic flavivirus RNA (sfRNA) interacts with G3BP1, G3BP2, and Caprin1 to regulate the translation of ISG mRNA. Viruses can escape immune response, which are conducive to the replication of DENV (90, 91).

When the body is infected with rubella virus (RUBV), it induces the synthesis of G3BP; however, SGs are not formed. In addition, G3BP exists alone in the form of particles and plays a role in the late stage of viral replication (92). Lindquist et al. stated that SGs are formed when cells are infected with respiratory syncytial virus (RSV). G3BP is a key molecule that affects the replication of RSV and virus replication is enhanced in cells that produce stress responses (95). G3BP1 is also necessary for the replication of the Newcastle disease virus (NDV) (93). When infected by the infectious bursal disease virus (IBDV), the expression of G3BP1 is enhanced, which induces the formation of a large number of SGs and promotes the replication of the virus in the host cell (86). The proteins G3BP1, TIAR and Caprin-1 in SGs are important for the replication of the Zika virus (ZIKV), and knockout of these proteins decrease the virus titer. The interactions between the ZIKV RNA and G3BP1 as well as those between the viral capsid protein and G3BP1 and Caprin-1 can cause damage to the assembly of SGs and promote viral replication (94).

By summarizing the relationship between the above-mentioned viruses, we inferred that the interactions between G3BP and SGs is complicated. For most viruses, G3BP exhibits antiviral effect and is cleaved by viral proteins to inhibit the formation of SGs. Although some G3BP proteins have antiviral effects, the formation of SGs during virus infection can promote the replication of the virus, such as MRV. Viruses can also use G3BP to promote its proliferation. In the process of promoting viral replication, the changes in SGs are different in different virus-infected cells. For example, the formation of SGs and viral replication are promoted during RSV infection, whereas the assembly of SGs is impaired during ZIKV infection, and SGs formation has no effect on viral replication during RUBV infection.

## G3BP Affects the Metabolism of mRNA

G3BP also has a function independent of SGs, which involves regulating the target mRNAs and controlling gene expression.

### G3BP1 Promotes or Stabilizes mRNA Expression

**Figure 3:**
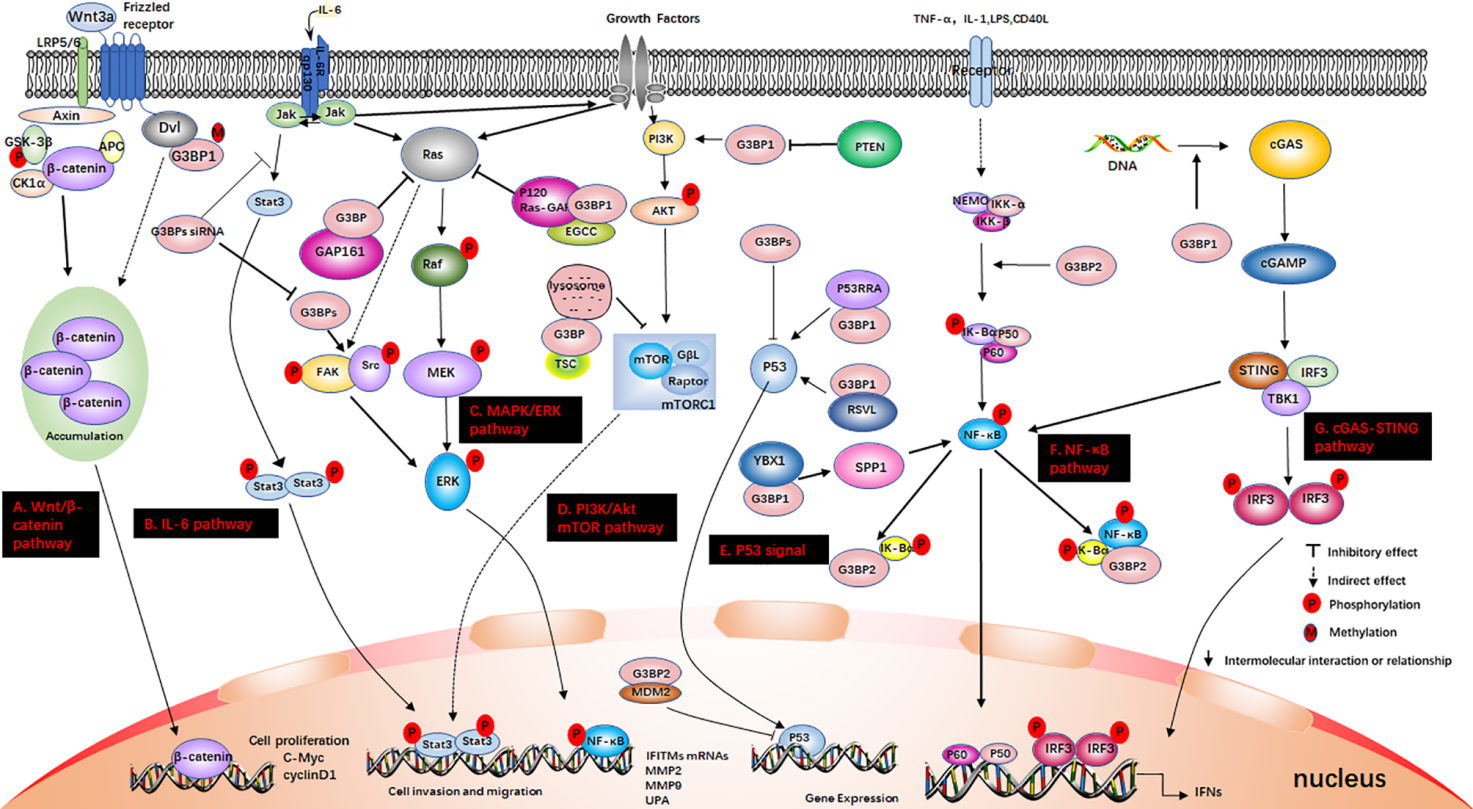
G3BP participates in some signaling pathways. **(A)** The Wnt/β-catenin pathway. Axin, and adenomatous polyposis coli (APC) activate glycogen synthase kinase‐3 beta (GSK-3β). This causes the degradation of β-catenin, which is mediated by proteasomes. Wnt3a activates this pathway. Ctnnb1 mRNA in the Dishevelled3 (Dvl3) complex has the ability to resist the degradation of β-catenin and can also stimulate the rapid accumulation of β-catenin in cells. This causes the translocation of β-catenin to the nucleus and stimulates the activation of genes. **(B)** IL-6 pathway. IL-6 binds to IL-6R and glycoprotein 130 (gp130) on the membrane, dimerizes gp130, and initiates intracellular signals. First, Janus kinase (JAK) is activated, and then STAT3 is phosphorylated to form a homodimer, which is transferred to the nucleus to induce the expression of target genes. IL-6 can also activate the Src, MAPK cascade and PI3K signaling pathway; **(C)** MAPK/ERK pathway. Growth factors bind to specific receptors on the cell membrane to form dimers. Ras dissociates from GDP and binds to GTP. Ras is first activated, and it further activates the serine/threonine protein kinase (Raf-1), which phosphorylates it, thereby activating MEK; **(D)** PI3K/Akt mTOR pathway. When growth factor binds to receptors, it can change the protein structure of Akt and further activate the downstream target molecule, mTOR. The tumor suppressor protein PTEN can dephosphorylate Akt and reduce its activation. It can also prevent downstream signal transduction, which is regulated by Akt. It is also a negative regulator of PI3K. **(E)** P53 signal. P53 is a tumor suppressor gene that can respond to various cell signals such as DNA damage, oncogene expression, nutrient deprivation, and ribosome dysfunction. P53 regulates the genome integrity, cell cycle arrest, and cell apoptosis; **(F)** NF-κB pathway. When the upstream signal factor binds to the receptor on the cell membrane surface, the receptor conformation changes, and the signal is transmitted to IKK. IκBα is phosphorylated under the action of IKK-α/β, and the phosphorylated IκBα is then ubiquitinated and degraded by the proteasome to release p50/p65. Subsequently, the p50/p65 dimer exposes the nuclear localization sequence (NLS), rapidly enters the nucleus from the cytoplasm, and combines with specific sequences on the nuclear DNA to promote the transcription of related genes; **(G)** cGAS-STING pathway. cGAS is an important cytoplasmic sensor for DNA and the cGAS-STING pathway is very important for the defense against viral infections. cGAS recognizes exogenous DNA and catalyzes the synthesis of cGAMP. Then, cGAMP binds to and activates STING, recruits TBK1, and phosphorylates IRF3 and activates it to induce the production of interferons.

G3BP1 regulates the stabilization and attenuation of mRNAs to regulate protein levels. G3BP1 is stimulated by the Wnt family member 3a (wnt3a) and the arginine of the RGG domain is methylated to promote the level of Ctnnbl mRNA (105) (**Figure 3**). However, G3BP1 inhibits the expression of peripheral myelin protein 22 (PMP22) mRNA, which plays a role in the regulation of breast cancer cell proliferation (106). The insulin-like growth factor-II mRNA-binding protein 1(IMP-1) binds to HuD (a member of the Elav protein family) and G3BP1 in an RNA-dependent manner and then directly binds to Tau mRNA to stabilize it. Tau is a microtubule-associated protein that is highly regulated during neuronal cell differentiation (107). G3BP1 can also negatively regulate the axon mRNA of mammalian neurons, which is related to neural differentiation and regeneration (108). G3BP1 interacts with cyclin-dependent kinase 7 (CDK7) to stabilize it, while the cyclin-dependent kinase 9 (CDK9) mRNA is degraded after binding to G3BP. The expression of CDK7 and CDK9 is regulated in an alternating manner to ensure the synthesis of full-length cardiac muscle mRNA (109). The expression of G3BP1 increases during cardiac hypertrophy. G3BP1 binds to the consensus sequence in the stem loop region of the microRNA (miR)-1-2 precursor to limit the processing of miR-1, increase the expression of eukaryotic translation initiation factor 4E (eIF4E) and promote the translation of RNA. Thus, G3BP1 plays an important role in the development of cardiac hypertrophy (110).

### G3BP1 Degrades mRNA

G3BP1 can cleave the 3’-untranslated region (3’-UTR) of cellular-myelocytomatosis (c-Myc) mRNA in a phosphorylation-dependent manner (18). G3BP1 is an endoribonuclease that requires phosphorylation sites to exert its catalytic activity. In proliferating cells, G3BP1 is hypophosphorylated, leading to the loss of its ability to cleave mRNA. In resting cells, G3BP1 is hyperphosphorylated, which helps it maintain its ability to cleave mRNA (40). G3BP is recruited to mRNA of binder of Arl two (BART) and degrades it (50). Cluster of differentiation 24 (CD24) binds to G3BP and blocks the cleavage of BART transcripts. This further affects the invasion and metastasis of pancreatic cancer cells (111). G3BP1 interacts with the coding mitochondrial H+-ATP synthase subunits (ATP5B, β-F1-ATPase) and inhibits its translation in mitochondria. So G3BP1 plays a role in glycolytic conversion (112). CLUH (clustered mitochondria homolog) can recruit G3BP1, G3BP2 and mTOR, thereby enhancing mitochondrial autophagy and inhibiting mitochondrial anabolic pathways (113).

### G3BP2 Regulates mRNA Levels

G3BP2 may also affect the stability and translation efficiency of specific mRNAs. It controls the mammary tumor-initiating cells and immune checkpoints. It regulates mammary tumorigenesis by stabilizing the levels of squamous cell carcinoma antigen recognized by T-cells 3 (SART3) mRNA (114) and the immune checkpoint molecule programmed death-ligand 1 (PD-L1) mRNAs (115) through the RNA binding motifs. G3BP2 is involved in the NF-κB signal transduction cascade, nuclear transport, and RNA metabolism. The NTF2 domain of G3BP2 is recognized by the cytoplasmic retention sequence (CRS) of IκBα, which interacts with the IκBα/NF-κB complex. Overexpression of G3BP2 promotes the retention of IκBα in the cytoplasm (116) (**Figure 3**).

G3BP1/2 may also be involved in the IFN regulatory system. G3BP1/2 can regulate the translation of interferon-induced transmembrane protein 1-3 (IFITM1-3) through the MAPK/ERK pathway or by interacting with the 3’-UTR of the (IFITM1-3), thereby resulting in the accumulation of proteins (104) (**Figure 3**). IFITM3 plays a role in inhibiting tumor development. The interaction between CHIKV nsP3 and G3BP1 effectively reduces the activity of G3BP1 in the translational regulation of its target gene IFITM3, resulting in downregulation of the IFITM3 protein. This can be used to develop new strategies to target the decomposition of SGs and aid in the treatment of diseases (117).

## G3BP and Disease

G3BP is related to the tumor development process, including promoting the entry of cancer cells into the S phase and enhancing their cell growth (118). The G3BP protein family is overexpressed in a variety of human tumors, and G3BP2 is overexpressed to a greater extent than G3BP1 (119). G3BP plays important roles in cell proliferation, differentiation, and apoptosis. It also participates in a variety of signal transduction pathways involved in carcinogenesis, including the NF-κB, ERK, p53, and Ras (**Figure 3**). Therefore, G3BP can be considered a novel target for cancer treatment (120).

### G3BP and Cancer

#### G3BP Regulates Breast Cancer

G3BP is involved in the development and metastasis of cancer through multiple pathways (121). The interaction of G3BP1 and GSK-3β inhibits the degradation of β-catenin in the cytoplasm and promotes the proliferation of breast cancer (122) and esophageal cancer by enhancing the stability of β-catenin (123). However, the literature suggests that G3BP1 negatively regulates the Wnt/β-catenin signaling pathway, in which the level of β-catenin decreases but the expression of G3BP1 increases (105). The mammalian target of rapamycin complex 1 (mTORC1) controls systemic metabolism, and its overexpression drives the growth and movement of breast cancer cells (124). G3BP binds to the tuberous sclerosis complex (TSC) to the lysosome and inhibits mTORC1 signaling, thereby inhibiting breast tumor cell migration (125) (**Figure 3**).

#### G3BP Regulates Kidney Cancer

G3BP1 contributes to the proliferation, migration and invasion of renal cell carcinoma (RCC), and knockdown of G3BP1 blocks the IL-6/STAT3 signaling and reduces the metastatic ability of RCC (126) (**Figure 3**). The Y-box binding protein 1 (YBX1) interacts with G3BP1 to upregulate the secreted phosphoprotein 1 (SPP1) and activate NF-κB, which ultimately promotes RCC metastasis (127). G3BP1 enhances the resistance of sunitinib-resistance RCC (128). This suggests that targeting G3BP1 may prove to be an effective therapeutic strategy for RCC (129).

#### G3BP Regulates Lung Cancer

Knockdown of G3BP in human lung cancer (H1299) cells inhibits the activation of Src, focal adhesion kinase (FAK) and ERK1/2 and decreases the expression of NF-κB. It also inhibits the expression levels of matrix metalloproteinase-2 (MMP-2), matrix metalloproteinase-9 (MMP-9), and urokinase-type plasminogen activator (uPA), contributing to the inhibition of the proliferation, invasion, and migration of lung cancer cells (130) (**Figure 3**). G3BP1 is required for the activation of the senescence-associated secretory phenotype (SASP) (131), and the effect of SASP on the proliferation and migration of cancer cells is very complicated (132).

#### G3BP and Tumor Suppressor Factors

G3BP binds to the tumor suppressor p53 both *in vivo* and *in vitro* and negatively regulates the expression of p53 (133) (**Figure 3**). However, in the presence of P53RRA in the cytoplasm, it interacts with G3BP1 to remove p53 from the G3BP1 complex. A small amount of G3BP1 may form a complex with p53, so that there may be more p53 in the nucleus. This can lead to cell cycle arrest, apoptosis, and hypertrophy (134). Resveratrol (RSVL) directly binds to G3BP1 and prevents the interaction of G3BP1/USP10, which enhances the USP10-mediated de-ubiquitination of p53, thereby increasing p53 expression (135). G3BP2 also interacts with murine double minute 2 (MDM2) to inhibit p53 by targeting it for proteasome degradation and translocation (133). The tumor suppressor gene *PTEN* acts on the phosphoinositide 3-kinase (PI3K) pathway and inhibits the expression of G3BP1 through its own phosphatase activity, thereby also inhibiting tumorigenesis (136). Epigallocatechin-3-gallate (EGCG), a polyphenolic compound which is the main catechin in green tea, interferes with the interaction of G3BP1 and RasGAP activator protein and further inhibits the activation of Ras and the occurrence of lung tumors (137). Zhang et al. identified a novel peptide, GAP161, which also binds to G3BP and inhibits the Ras signaling pathway, thereby providing a new strategy for cancer therapy (138).

#### G3BP and Other Diseases

The G3BP1/RIG-I/MAVS relay is a component of the Wnt signaling pathway. G3BP1 is a known regulator of atherosclerosis, and targeting this relay may help reduce atherosclerosis (57). Inactivation of the gene encoding G3BP leads to embryonic death and growth retardation (139). The zebrafish G3BP1 and human G3BP1 orthologs exhibit 67.8% sequence identity. Inhibition of G3BP1 will interfere with neuronal development in zebrafish (125). G3BP1 interacts with p62 and USP10 to inhibit protein aggregation. Hence, it controls the toxicity of ubiquitinated proteins by controlling their protein aggregation. Therefore, G3BP1 can be used as a therapeutic target for ubiquitinated protein aggregation-related diseases, such as Parkinson’s disease (PD) and cystic fibrosis (CF) (140).

## Concluding Remarks and Further Perspectives

G3BP is modified under different stress conditions to form SGs. It does not perform a single specific function in cells; however it can respond to a series of signals and control gene expression in various manners. It is involved in the formation of SGs, mRNA metabolism, and cell cycle regulation (18, 46, 118). In addition, it participates in the regulation of some signaling pathways, including the Wnt/β-catenin (105), NF-κB (116), ERK/MAPK (104), JAK/STAT (126), PI3K/Akt mTOR pathway (125). It is also associated with the formation of tumors, embryonic development, and neurological diseases.

Viruses can use G3BP to promote their replication and evade the cellular immunity of the host. Some viruses target G3BP by disrupting the formation of SGs or controlling the translation of ISGs and interact with G3BP to achieve viral replication and evade the natural immunity of the host. However, some issues still that need to be addressed. (1) G3BP supports the replication of some viruses, whereas in others, it plays an antagonistic role. Therefore, its mechanism of action needs to be studied further; (2) whether G3BP is related to protein chaperones and cell types in regulating the attenuation and stability of mRNA remain unclear; and (3) G3BP is a novel therapeutic target that can be used to develop new strategies for cancer treatment, which warrants further research.

## Author Contributions

Conceptualization, WK and DL. Writing—original draft preparation, WK, YW, WY, JZ, and DL. Writing—review and editing, WK, YW, and WY. Supervision, HZ and DL. Validation, all authors. All authors contributed to the article and approved the submitted version.

## Funding

This work was supported by Grants from Key Tasks of Lanzhou Veterinary Research Institute, Chinese Academy of Agricultural Sciences (CAAS-ASTIP-2020-LVRI-01), National Natural Science Foundation of China (31941002).

## Conflict of Interest

The authors declare that the research was conducted in the absence of any commercial or financial relationships that could be construed as a potential conflict of interest.

## Publisher’s Note

All claims expressed in this article are solely those of the authors and do not necessarily represent those of their affiliated organizations, or those of the publisher, the editors and the reviewers. Any product that may be evaluated in this article, or claim that may be made by its manufacturer, is not guaranteed or endorsed by the publisher.
